# Engineering of *g*-C_3_N_4_ for Photocatalytic Hydrogen Production: A Review

**DOI:** 10.3390/ijms25168842

**Published:** 2024-08-14

**Authors:** Yachao Yan, Qing Meng, Long Tian, Yulong Cai, Yujuan Zhang, Yingzhi Chen

**Affiliations:** 1 School of Materials Science and Engineering, University of Science and Technology Beijing, Beijing 100083, China; m202210388@xs.ustb.edu.cn (Y.Y.); m202310345@xs.ustb.edu.cn (Q.M.); m202321707@xs.ustb.edu.cn (L.T.); m202321698@xs.ustb.edu.cn (Y.C.); 2 Shunde Graduate School, University of Science and Technology Beijing, Foshan 528399, China

**Keywords:** *g*-C_3_N_4_, photocatalyst, hydrogen generation, modification engineering

## Abstract

Graphitic carbon nitride (*g*-C_3_N_4_)-based photocatalysts have garnered significant interest as a promising photocatalyst for hydrogen generation under visible light, to address energy and environmental challenges owing to their favorable electronic structure, affordability, and stability. In spite of that, issues such as high charge carrier recombination rates and low quantum efficiency impede its broader application. To overcome these limitations, structural and morphological modification of the *g*-C_3_N_4_-based photocatalysts is a novel frontline to improve the photocatalytic performance. Therefore, we briefly summarize the current preparation methods of *g*-C_3_N_4_. Importantly, this review highlights recent advancements in crafting high-performance *g*-C_3_N_4_-based photocatalysts, focusing on strategies like elemental doping, nanostructure design, bandgap engineering, and heterostructure construction. Notably, sophisticated doping techniques have propelled hydrogen production rates to a 10^4^-fold increase. Ingenious nanostructure designs have expanded the surface area by a factor of 26, concurrently extending the fluorescence lifetime of charge carriers by 50%. Moreover, the strategic assembly of heterojunctions has not only elevated charge carrier separation efficiency but also preserved formidable redox properties, culminating in a dramatic hundredfold surge in hydrogen generation performance. This work provides a reliable and brief overview of the controlled modification engineering of *g*-C_3_N_4_-based photocatalyst systems, paving the way for more efficient hydrogen production.

## 1. Introduction

The world is facing critical environmental challenges and energy problems including air/water pollution, global warming, and energy resources shortage, as a result of increased population, industrialization, and energy consumption [1]. In this regard, a shift in the world’s energy focus from traditional fossil fuels to sustainable and renewable energy is imminent. Green hydrogen energy is one of the most promising alternative energy sources. Hydrogen can successfully achieve zero carbon emissions, with a clean and non-toxic combustion process that emits no nitrogen oxides and the only product is water vapor [2]. Moreover, the energy yield of hydrogen is about 122 kJ/g, which is 2.75 times higher than hydrocarbon fuels [3]. Nevertheless, economic and clean hydrogen production is the fundamental point of much research. Currently, the process of hydrogen production generates CO_2_ byproducts [4]. Photocatalytic hydrogen production utilizing solar energy on the catalyst surface is a feasible circumvention method. Semiconductor photocatalysts are water redox mediators based on electron–hole pairs that play an auxiliary role in the water-splitting reaction and are not converted or consumed in the procedure. The huge solar to H_2_ efficiency and easy operation approach make it truly a viable option for hydrogen production.

Tremendous efforts were devoted to exploiting high-performance photocatalysts, which can be categorized into two groups: organic and inorganic semiconductors. Large amounts of inorganic semiconductors were investigated in water splitting, including metal oxides (TiO_2_, Fe_2_O_3_, BiVO_4_, NiO, WO_3_) [4,5,6,7,8], metal nitrides (Ta_3_N_5_, TaON) [9], metal sulfides (CdS, MoS_2_, In_2_S_3_) [10], complex metal semiconductors (BN/GdTiO) [11], and metal-free semiconductors (B-C-N alloy) [12]. Given that semiconductor photocatalysts consisting of earth-abundant and inexhaustible elements cater to the large-scale industrial application of H_2_ production, organic semiconductors are more economically viable promoters. To date, various kinds of organic semiconductors have been applied in the photocatalytic hydrogen evolution reaction (HER), for instance, polymers such as perdodiimide (PDI) [13], porphyrin (Pors) [14], and polypyrrole (PPY) [15]; carbon nanotubes such as fullerene (C_60_) [16], porous crystals such as metal-organic frameworks (MOFs) and covalent organic frameworks (COFs) [17]; and organic polymers [18]. Organic nanostructure semiconductors have become a rising star for photocatalytic hydrogen production, possessing high ultraviolet–visible absorption spectra (UV–vis), high charge separation efficiency, and abundant reaction sites for various applications.

Among many organic nanostructure semiconductors, graphitic carbon nitride (*g*-C_3_N_4_) stands out for its chemical and thermal stability, non-hazardous nature, cost-effectiveness, and the simplicity of its device fabrication process. These attributes make *g*-C_3_N_4_ an excellent candidate for engineering applications, particularly in the field of photocatalysis. Its narrow band gap of 2.7 eV endows it with the capability to absorb visible light efficiently, positioning it as a strong contender for photocatalytic hydrogen generation [18]. The significance of engineering in the context of *g*-C_3_N_4_ lies in enhancing its photocatalytic performance to meet practical industrial and environmental needs. Despite its promise, the photocatalytic efficiency of pristine *g*-C_3_N_4_ is limited due to challenges such as a high recombination rate of photoexcited charge carriers and a low quantum efficiency [19]. Addressing these issues is crucial for the advancement of *g*-C_3_N_4_ in real-world applications. The photocatalytic H_2_ generation occurring at the *g*-C_3_N_4_/water interface is highly dependent on several factors, including the size, morphology, and defects of *g*-C_3_N_4_. It is essential to tailor these properties through synthesis of specific structures, morphologies, and compositions. Techniques such as nanostructure modulation, creation of hollow structures, doping with cocatalysts, and formation of composites can alter particle size, structure, specific surface area, and surface functionality. These modifications also have the potential to extend the light-harvesting capabilities of *g*-C_3_N_4_, thereby significantly improving its visible-light photocatalytic performance [20,21]. Scientists are exploring various strategies to achieve these objectives, by controlling its micro/nanostructure and energy band structures to optimize its photocatalytic properties.

In this review, the latest rational modification strategies were investigated to enhance the photocatalytic performance of *g*-C_3_N_4_ for H_2_ generation under visible light irradiation, including elemental doping to modify the electronic structure and improve charge separation, nanostructure design to facilitate better charge transport and light absorption, band gap engineering to optimize solar spectrum absorption and refine the photocatalytic process, and heterostructure construction to form heterojunctions that promote charge separation and suppress recombination. By elucidating these strategies, the review provides a brief overview of the engineering efforts directed toward overcoming the limitations of *g*-C_3_N_4_ and highlights the potential pathways for its future development and application in sustainable energy production and environmental remediation.

## 2. Principles of Photocatalytic Hydrogen Production

Photocatalytic water splitting involves electrochemical reactions and photophysical mechanisms and is widely used in organic semiconductor photocatalysts to produce hydrogen energy. The water splitting process is a thermodynamic uphill reaction, with the Gibbs free energy of 1.23 eV, as shown in Equation (1). Since the standard reduction potential of water is 0 V (H_2_O/H_2_) and the standard oxidation potential is 1.23 V (H_2_O/O_2_), the electrolysis of a molecule of water into hydrogen and oxygen in an electrolytic cell requires an energy of 1.23 eV, as shown in Equations (2)–(4) [22]. To achieve water decomposition, the band gap of the photocatalyst should be greater than 1.23 eV, and the wavelength of the absorption side should be less than 1000 nm. Due to the presence of overpotential, the band gap of the catalyst is generally greater than 1.8 eV [23]. If the reaction is carried out in the visible range (λ > 400 nm), the band gap of the photocatalyst should be less than 3.0 eV [24]. In addition, to achieve reasonable solar conversion efficiency, a band gap of less than 2.2 eV is recommended. Thus, the optimal bandgap should be between 1.8 eV and 2.2 eV.

(1) $$H_{2}O\to H_{2}+1/2O_{2};∆G^{0}=1.23\;eV$$

(2) $$E^{0}=∆G/nF$$

(3) $$2H^{+}+2e^{-}\to H_{2}$$

(4) $$H_{2}O+2h^{+}\to 1/2O_{2}+{2H}^{+}$$

**Figure 1:**
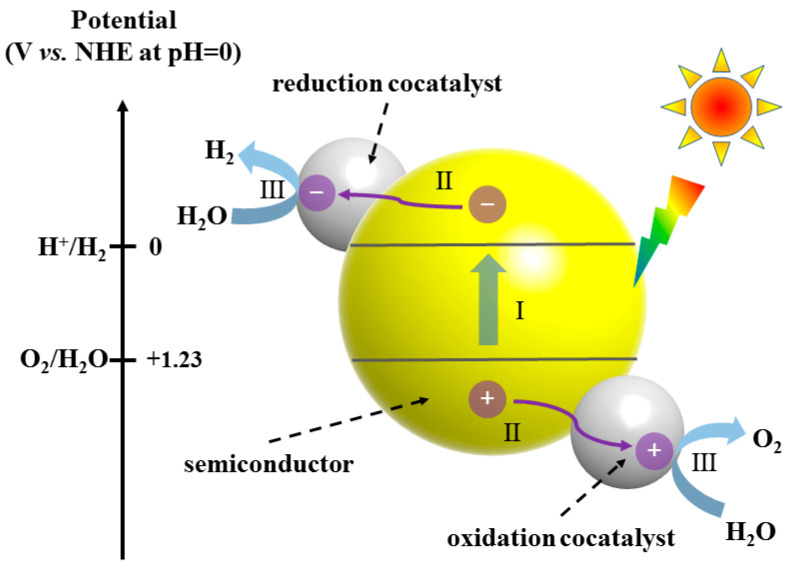
Photocatalytic H_2_ production of semiconductor.

Generally, excellent photocatalytic hydrogen production efficiency is not only affected by band structure and location but also depends on kinetic factors such as charge transfer, separation and surface redox reactions. The photocatalytic process can be divided into four steps: photon absorption, photoexcited charge separation, charge diffusion/transport, and catalytic reaction at the active site of the catalyst [25] (Figure 1). Under illumination, the photocatalyst with a band gap between 1.8 and 2.2 eV enters the conduction band side by excitation electrons, leaving holes on the valence band side, and creating electron–hole pairs. Subsequently, the carriers will migrate from the body of the photocatalyst to the surface of the catalytic gas generation. During this process, the electron–hole pair may recombine due to very small band gap energy and short lifetime, which will severely limit the number of effective carriers reaching the surface of the material, thus affecting the final gas precipitation performance. Finally, the H_2_O molecules adsorbed on the photocatalyst’s surface are released along with the electrons (holes) accumulated there. Through a redox reaction, the photogenerated holes catalyze the oxidation of water into O_2_ and H^+^, while the photogenerated electrons facilitate the reduction of H^+^ to H_2_. This reaction results in the production of H_2_ and O_2_, thereby completing the entire photochemical process. This step is fundamentally dependent on the nature of the photocatalyst chemical agent (nature of the active site), reaction conditions, and adsorption of reactants [26]. Therefore, to improve the efficiency of the photolysis of water, it is necessary to thoroughly consider many factors affecting the efficiency of photolysis of water.

In photocatalysis, due to the small energy of the band gap, if the electrons and holes do not reach the substrate immediately, the photogenerated electron–hole recombines and releases heat energy, reducing the H_2_ yield. In addition, the surface reverse reaction (SBR) of photogenerated H_2_ and O_2_ also leads to a decrease in H_2_ yield. In addition, the thermal equilibrium of excitons generated by photoexcitation with their surroundings usually occurs very fast (less than 10 ps), and the rapid electron–hole recombination reaction during the relaxation of electrons from the high excited state to the lowest excited state always involves a large amount of energy loss [27]. Therefore, well-designed semiconductor-based photocatalysts are required to achieve efficient charge separation and meet the activation energy required for non-spontaneous reactions under solar irradiation. In recent years, researchers have made a series of substantial progress in the application of photocatalysts in hydrogen production through strategies such as element doping, surface modification, morphology control, heterostructure construction, and cocatalyst deposition, but there are still problems such as poor photostability and limited photocatalytic activity of photocatalysts [28], resulting in most photocatalytic systems being unable to meet the actual needs of hydrogen production applications. *g*-C_3_N_4_ has a suitable and stable bandgap (2.7 eV) and excellent thermal and chemical stability, making it a promising candidate in the field of photocatalytic hydrogen production. Therefore, the researchers tried to improve the photocatalytic performance of *g*-C_3_N_4_ by improving/combining multiple strategies to meet the practical application needs of *g*-C_3_N_4_ in the field of hydrogen production. Recently, Qureshi [29] et al. combined a porous metal-organic framework (MOF), Ni-MOF-74 and B-doping to construct a novel B-doped *g*-C_3_N_4_/Ni-MOF-74 porous heterojunction, which increased the hydrogen evolution rate of *g*-C_3_N_4_ to 2190 μmol·g^−1^·h^−1^, which is almost 11 times that of BCN. Liu [30] et al. used a supercritical CH_3_OH (ScMeOH) post-processing strategy to modulate the structure of *g*-C_3_N_4_ to convert a partial phase transition of *g*-C_3_N_4_ into carbon nitride with a poly(heptazinyl imide)-like structure (Q-PHI) as well as abundant methyl and hydroxyl groups. The improved *g*-C_3_N_4_ exhibited excellent photocatalytic hydrogen evolution activity, and the hydrogen evolution rate was 7.2 times that of the original *g*-C_3_N_4_. Sun [31] et al. designed a novel polymeric-inorganic *g*-C_3_N_4_ nanosphere-CdZnS heterojunction photocatalyst, and the optimized composite exhibited an enhanced H_2_ release rate of 582.3 μmol·g^−1^·h^−1^, which was 3.7 times that of the original *g*-C_3_N_4_. Wang [32] et al. deposited non-precious metal CoP/CoO cocatalysts on the surface of *g*-C_3_N_4_ to make the photocatalyst exhibit a stable hydrogen evolution rate of 860 μmol·g^−1^·h^−1^, which was 30 times and 1.5 times higher than the evolution rates of CoO/*g*-C_3_N_4_ and Pt/*g*-C_3_N_4_, respectively.

## 3. Structure and Properties of *g*-C_3_N_4_

**Figure 2:**
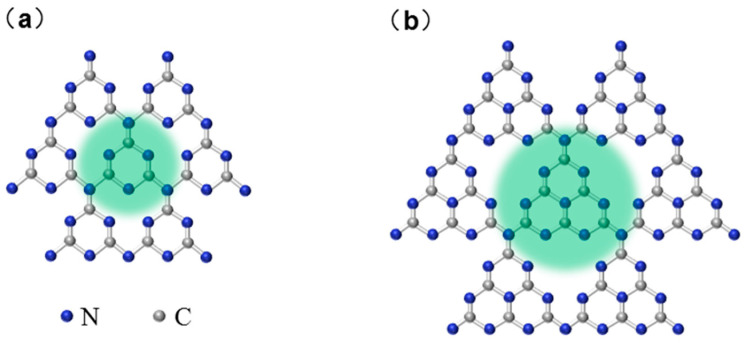
Molecular structure of *g*-C_3_N_4_: (**a**) triazine structure (C_3_N_3_); (**b**) 3-s-triazine structure (C_6_N_7_).

*g*-C_3_N_4_ is a type of two-dimensional semiconducting polymer that has a 2D layered structure similar to graphene. *g*-C_3_N_4_ possesses two basic building blocks, one is a triazine structure (C_3_N_3_) and the other is a 3-s-triazine structure (C_6_N_7_) [33], both of which behave as allotropes, as shown in Figure 2. The inter-ring bridges of the above structures are connected by nitrogen atoms. The C and N atoms making up the basic unit of *g*-C_3_N_4_ form a network structure, as a result of sp^2^ hybridization, forming a highly delocalized π conjugated system [34]. The unique physicochemical structure gives *g*-C_3_N_4_ remarkable properties: excellent thermal and chemical stability, tunable electronic structure, biocompatibility, and facile low-cost synthesis procedure. Resembling the structure of graphene, the presence of weak van der Waals forces in *g*-C_3_N_4_ interlayer connections results in the arrangement of atoms in each layer into a honeycomb structure with strong covalent bonds [35], which confers excellent thermal stability (up to 600 °C) and chemical stability (resistance to strong acids and bases) [36]. In addition, *g*-C_3_N_4_ has a high stability band gap (*E*_g_ = 2.7 eV); therefore, it can meet the potential requirements of the photocatalytic redox reaction. The special visible light absorption band overcomes the limitations of the narrow spectral response range of conventional photocatalysts (λ = 460–475 nm), and this band structure enables *g*-C_3_N_4_ to generate photogenerated electrons and holes, which can be investigated as promising candidates for photocatalytic applications, particularly hydrogen and oxygen production [37]. Despite its optimal band structure, challenges remain for its widespread adoption in the long run, owing to the insufficient visible light (<470 nm) harvest, slow rate charge mobility from bulk to surface, fast recombination rate, and poor adsorption and activation of water by surface structure [38]. These limitations are related to poor crystallinity, visible light capture at the edges, easy recombination of charge pairs, small specific surface area, and slow charge shift [39]. Organic semiconductors are characterized by structural diversity and synthetic functionality, so these limitations can be overcome by tuning optoelectronic properties through structure/performance engineering.

In recent years, efforts have been made to improve the photocatalytic performance of *g*-C_3_N_4_, such as chemical doping, morphology, and heterostructure formation [34]. *g*-C_3_N_4_ possesses a broadband spectrum with a lone pair valence band (sp^2^-N bond), which could act as a potential coupling with various functional materials. Depending on the photocatalytic process, *g*-C_3_N_4_ nanocomposites consist of several primary structural mechanisms: namely, *g*-C_3_N_4_ based complex system, *g*-C_3_N_4_ based metal-free heterojunction, *g*-C_3_N_4_/metal oxide (metal sulfide), *g*-C_3_N_4_/noble metal heterostructures, and *g*-C_3_N_4_/halide heterojunction [40,41]. Thanks to the two-dimensional layered structure, maximum hybridization of *g*-C_3_N_4_ with other components was ensured. For instance, various strategies were developed to improve photocatalytic activities of *g*-C_3_N_4_ particularly visible light, as an illustration of surface coupling hybridization through graphene, constructing mesoporous structures [42]. Very recently, benchmark studies have shown promising potential with doping metal/non-metal types, such as Fe, Ag Au, Pd, etc., and organic dyes. The versatility of crystal structure in the coalescence enables the heterostructure to enhance the quantum efficiency of the photocatalyst significantly. Moreover, *g*-C_3_N_4_ is synthesized from inexpensive raw materials such as dicyandiamide, melamine, thiourea, and cyanamide by a one-step polymerization method, which is abundant in content and convenient to synthesize, and its surface activity can be controlled without significantly affecting its theoretical structure and composition. Nevertheless, previous documents unambiguously reveal the dependable strategies to prepare desired tunable *g*-C_3_N_4_-based photocatalysts with significantly enhanced physio-chem characteristics and high photocatalytic activity performance.

## 4. Preparation of *g*-C_3_N_4_

**Figure 3:**
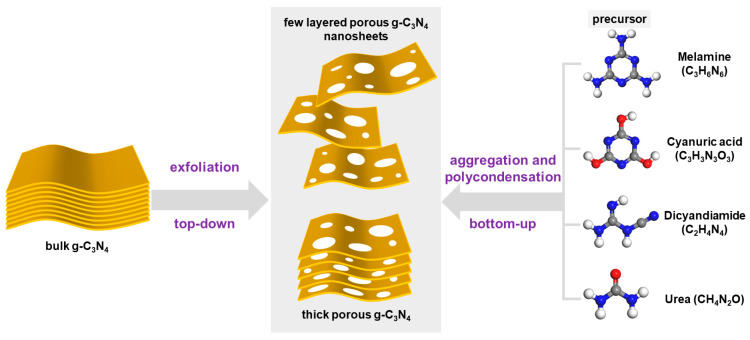
Schematic diagram of synthesis strategies *g*-C_3_N_4_ (gray, blue, white and red balls denote C, N, H and O atoms, respectively).

Currently, various preparation methods for *g*-C_3_N_4_ have been explored. These synthetic strategies can be broadly categorized into two types: “top-down” and “bottom-up” synthesis strategies [43]. The top-down synthesis strategy primarily involves subtracting from bulk *g*-C_3_N_4_ to obtain porous *g*-C_3_N_4_ or ultra-thin layered *g*-C_3_N_4_. The bottom-up synthesis strategy primarily involves the accumulation of precursor monomer molecules to synthesize *g*-C_3_N_4_. We constructed schematic diagrams (Figure 3) to facilitate the understanding of these two strategies.

**Figure 4:**
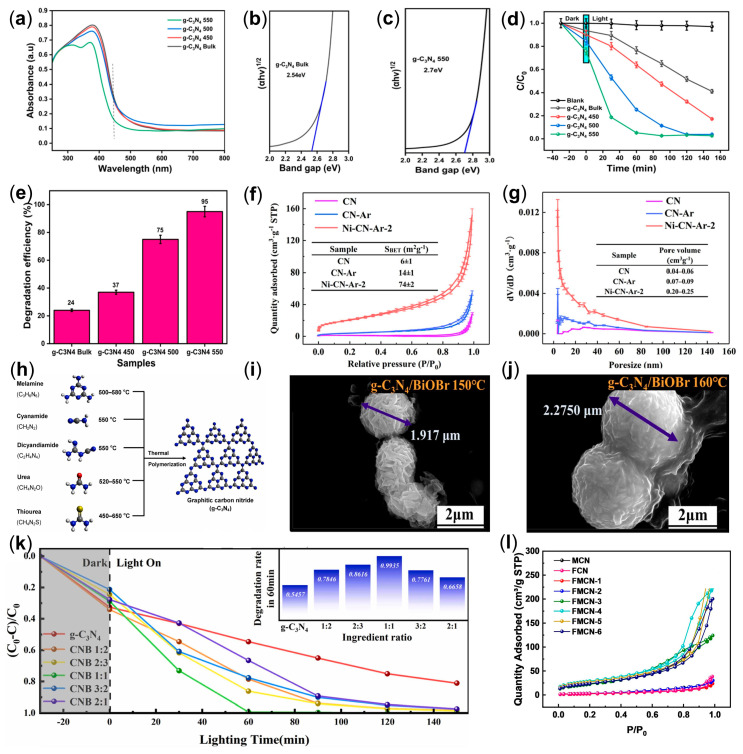
(**a**) UV–Visible absorbance spectra of *g*-C_3_N_4_ samples; band gap analysis-Tauc plot of (**b**) bulk *g*-C_3_N_4_ and (**c**) prepared *g*-C_3_N_4_; (**d**)time-dependent degradation kinetics; (**e**) photocatalytic degradation efficiency after 60 min of degradation [45]; (**f**) N_2_ adsorption–desorption isotherms; (**g**) pore size distributions (insert: detail structural parameters) [46]; (**h**) schematic illustration of the synthesis process of *g*-C_3_N_4_ by melamine, cyanamide, dicyanamide, urea, and thiourea [47]; SEM images of *g*-C_3_N_4_/BiOBr prepared at (**i**) 150 °C and (**j**) 160 °C; (**k**) degradation curves and 60 min degradation rates of samples prepared with different composition ratios [48]; (**l**) N_2_ adsorption–desorption isotherms of *g*-C_3_N_4_ [49]. Adapted with permission.

The core of the top-down synthesis strategy lies in disrupting the interlayer intermolecular forces to reduce thickness or form porous structures, thereby enhancing the quantum efficiency of light. The main methods include thermal exfoliation, gas exfoliation, liquid exfoliation, ultrasonic exfoliation, and combined exfoliation techniques [44]. In the latest research, Ganesan and colleagues prepared *g*-C_3_N_4_ with a high specific surface area (48.203 m²/g) through static air thermal exfoliation. It demonstrated excellent adsorption and photocatalytic degradation efficiency (Figure 4a–e) under ultraviolet light irradiation for the most common textile dye solutions such as methylene blue (MB), methyl orange (MO), and rhodamine B (RhB), with degradation efficiencies of 92 ± 0.18%, 93 ± 0.31%, and 95 ± 0.4%, respectively [45]. In another study, Ni single-atom doped *g*-C_3_N_4_ was innovatively prepared through a metal vapor phase exfoliation method to produce ultra-thin nanosheets [46]. The metal vapor generated by high-temperature calcination in an argon atmosphere disrupted the van der Waals forces between the layers of the two-dimensional *g*-C_3_N_4_, resulting in single-atom doped ultra-thin nanosheets. The Ni-CN-Ar catalyst prepared by the metal vapor phase exfoliation method exhibited excellent CO_2_ activity due to the synergistic effect of Ni single atoms and nanosheets, showing superior reduction performance (Figure 4f,g).

The bottom-up synthesis strategy involves the polymerization of precursor monomers, a method that can effectively transfer the specific functional structures of the precursors to the target *g*-C_3_N_4_. This is also a widely adopted strategy at present. The main methods include solvothermal synthesis and template-assisted methods, among others. The most commonly used precursors at present are melamine, cyanamide, dicyanamide, urea, and thiourea [47], with corresponding synthesis conditions, as shown in Figure 4h. This strategy can fully utilize the structural and functional group characteristics of the precursors. Recently, in the research by Zhao and colleagues, g-CN/BiOBr was synthesized using a solvothermal method, which resulted in the uniform encapsulation of layered g-CN around the flower-like spherical BiOBr structure (Figure 4i,j) [48]. By controlling the temperature and component ratios during the solvothermal process, the micromorphology of g-CN/BiOBr was regulated, effectively enhancing the photocatalytic activity (with an elimination rate of RhB as high as 99.74%) (Figure 4k). Additionally, using melamine as the raw material and utilizing silica sol and F127 as templates, a new method combining soft and hard template organization was proposed [49]. The research also found that the introduction of F127 not only incorporated carbon but also created nitrogen vacancies in *g*-C_3_N_4_. The prepared FMCN-4 achieved the maximum specific surface area of up to 104.18 m²/g (Figure 4l).

## 5. Modification Strategies over *g*-C_3_N_4_

### 5.1. Element Doping

As a fascinating conjugated polymer, *g*-C_3_N_4_ is a widely accepted paradigm with metal-free and visible light-responsive photocatalysts. *g*-C_3_N_4_ possesses a controllable tunable band gap [50]; therefore, its band structure can be adjusted by element doping to improve its photocatalytic and light absorption capabilities. Elemental doping can endow *g*-C_3_N_4_ with diverse morphologies, and modify the electronic structure and surface properties of *g*-C_3_N_4_ through orbital hybridization [51], to achieve a high degree of delocalization of π electrons in the living sites and conjugated structures on the surface of the photocatalyst, expand the light absorption capacity of *g*-C_3_N_4_, and promote the effective separation of photogenerated carriers, bestowing *g*-C_3_N_4_ superior photocatalytic performance. In recent years, metal doping, non-metal doping and co-doping strategies have been frequently exploited to tune the physiochemical properties such as achieving high activity, selectivity, improved visible light absorption, and desired engineered structures [52]. Table 1 summarizes the commonly used elemental doped *g*-C_3_N_4_ photocatalysts and their corresponding photocatalytic hydrogen evolution (PHE) properties in recent years.

#### 5.1.1. Metal Doping

**Figure 5:**
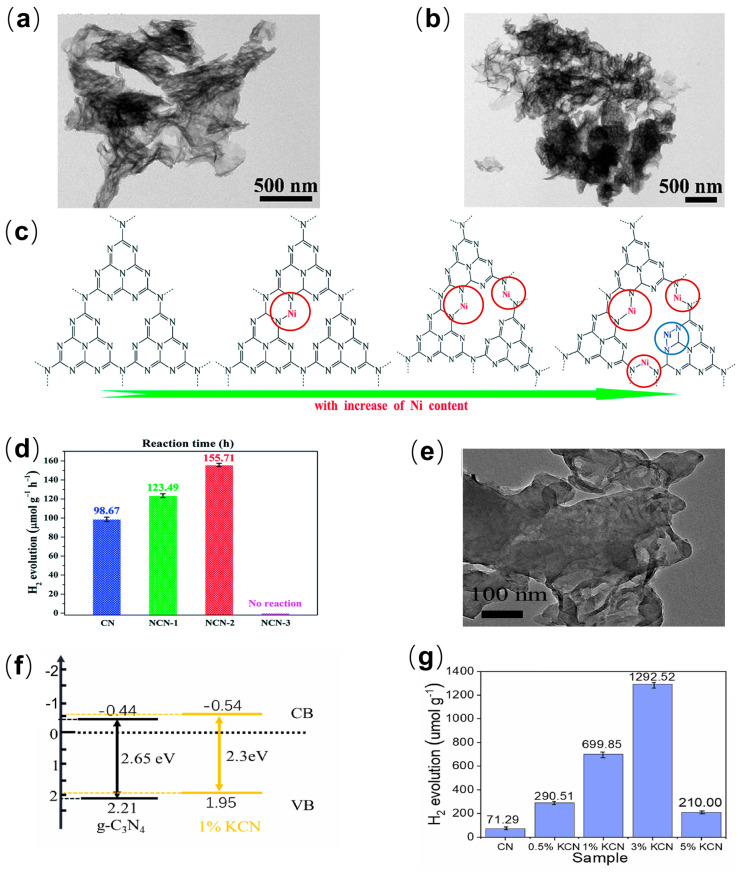
TEM images of (**a**) *g*-C_3_N_4_ and (**b**) NCN-x; (**c**) doping process for the NCN-x with an increase in Ni content; (**d**) photocatalytic hydrogen evolution of NCN-x [54] (adapted with permission); (**e**) TEM images of KCN; (**f**) band gap illustration of *g*-C_3_N_4_ and KCN; (**g**) photocatalytic H_2_ production rate of KCN [59] (adapted with permission).

Metal doping binds metal cations to *g*-C_3_N_4_ structures through weak linkages, and there is a strong interaction between metal cations and electron-rich sp^2^ nitrogen [77], resulting in the formation of strongly hybrid materials. Metal ions often introduce lattice defects and new impurity energy levels in *g*-C_3_N_4_, resulting in changes in the electronic band structure of *g*-C_3_N_4_ [78], thereby expanding the photoresponse range and effectively controlling electron–hole separation. Metal-doped *g*-C_3_N_4_, especially transition metals such as Fe^2+^, Co^2+^, Ni^2+^, Cu^2+^ [79], etc., were shown to be effective in adjusting the light absorption range, reducing the band gap and redox reaction potential, to improve photocatalytic performance. Ni^2+^ has emerged as a promising structural tuned metal-N coordination. Modification with the *g*-C_3_N_4_ conduction band was shown sensitive to the separation of photogenerated carriers. Deng [54] et al. developed a novel one-step pyrolysis route by adding Nickel formate to polymerization precursors (urea) to achieve high-level in situ Ni^2+^ doped of *g*-C_3_N_4_ nanosheets. The Ni-doped NCN-x samples possess a porous fold and layered nanosheet structure with coiled edges (Figure 5a) similar to the undoped sample (Figure 5b), indicating that the Ni modification has little effect on the texture of the *g*-C_3_N_4_ polymer. The Ni atom changes the valence band and the conduction band position of *g*-C_3_N_4_ in the form of the N atom coordination. Based on the difference in work function, Ni atoms tend to be embedded in the voids of the *g*-C_3_N_4_ frame at low levels of Ni doping, whereas at high levels N-Ni-N coordination bonds established in uncondensed amino nitrogen atoms (Figure 5c), which could become pivotal in the modification of *g*-C_3_N_4_ with improved band structure and photocatalytic performance. Because of the strong interaction between Ni and *g*-C_3_N_4_, the introduction of nickel ions can lead to superior absorption of visible light, reduce the band gap, and promote the separation of photogenerated carriers. With the appropriate concentration of Ni^2+^, the band gap of *g*-C_3_N_4_ was reduced from 2.73 eV to 2.45 eV, and the hydrogen production rate can reach 155.71 μmol·g^−1^·h^−1^, which is about 1.57 times that of the pure *g*-C_3_N_4_ (Figure 5d). In addition, the Ni-doped *g*-C_3_N_4_ photocatalyst also exhibited excellent photochemical stability and hydrogen release durability, and the hydrogen production rate was not significantly reduced after three photocatalytic hydrogen production experimental cycles (up to 30 h). The excellent photocatalytic cycle stability makes it practical in the field of hydrogen production.

In addition to transition metals, alkali metals are considered to be a viable alternative as well. For instance, the N-side coordination of K^+^ [59] and Na^+^ [58] with the *g*-C_3_N_4_ frame can significantly improve the transfer of the charge carrier while providing a tunable band gap. For the first time, Song [59] et al. used a combination of the mechanical chemical pre-reaction method and the two-step thermal polymerization reaction to successfully dope K^+^ into the *g*-C_3_N_4_ layer, forming superior thin (1–2 nm) K/*g*-C_3_N_4_ nanosheets (KCN). Compared with pure *g*-C_3_N_4_ nanosheets, KCN showed a thinner and porous morphological structure (Figure 5e) [80]. The superior thin structure and trace K doping made the *g*-C_3_N_4_ nanosheets possess a larger specific surface area and more surface defects. The refinement of K on the morphology of *g*-C_3_N_4_ is conducive to the increase in the reactive active site, which expanded the light absorption of *g*-C_3_N_4_ and improved the photocatalytic efficiency. Moreover, the doping of K narrowed the band gap of *g*-C_3_N_4_, making it easier for the charge to transfer to the reaction position in the layer. At the appropriate K^+^ concentration, the band gap of KCN was reduced from 2.65 eV to 2.3 eV, and it had a more negative conductive belt position (Figure 5f) so that the KCN hydrogen generation rate could reach 1292.52 μmol·g^−1^·h^−1^, which was about 18.13 times that of the pure *g*-C_3_N_4_ (Figure 5g). This was attributed to the fact that the maximum displacement of the conduction band made it easier for the electrons in the conduction band to be reduced, favoring photocatalytic hydrogen production. And the photocatalytic hydrogen evolution efficiency of KCN did not decrease significantly during the four cycles of 8 h, which proved that the doped catalyst had good stability. Its excellent photocatalytic activity and stability give it great potential in hydrogen production applications. Moreover, alkali–metal doping, and other wide-use elements, such as Zn, Li, V, Ag and Pd, etc. [81,82,83,84], can potentially improve the optoelectronic properties of *g*-C_3_N_4_. Because of strong interactions between metal cations and anions of N atoms that are attributed to lone pairs of electrons between the N side of *g*-C_3_N_4_, they show great potential for capturing metal cations.

#### 5.1.2. Non-Metal Doping

Recently, non-metal doping has emerged as a host material to maintain the metal-free property of *g*-C_3_N_4_, encouraged by the non-metallic doped elements including N [63], O [69], P [65], S [67], C [61], B [68] and halogen atoms, which constitute new and different bonding properties, simultaneously reducing the band gap and prolonging the mobility of photogenerated electrons. Moreover, non-metals possess high redox activity (e.g., selenium (Se), tellurium (Te), hydrogen bonding (e.g., nitrogen, oxygen, and sulfur), high ionization energies (proton transfer or protonation–deprotonation reactions), and high electro negativity (e.g., oxygen and fluorine) that can be leveraged as catalysts for electron transfer. Thus, non-metal catalysts can be further optimized by selecting the desired supporting material and the reaction conditions.

**Figure 6:**
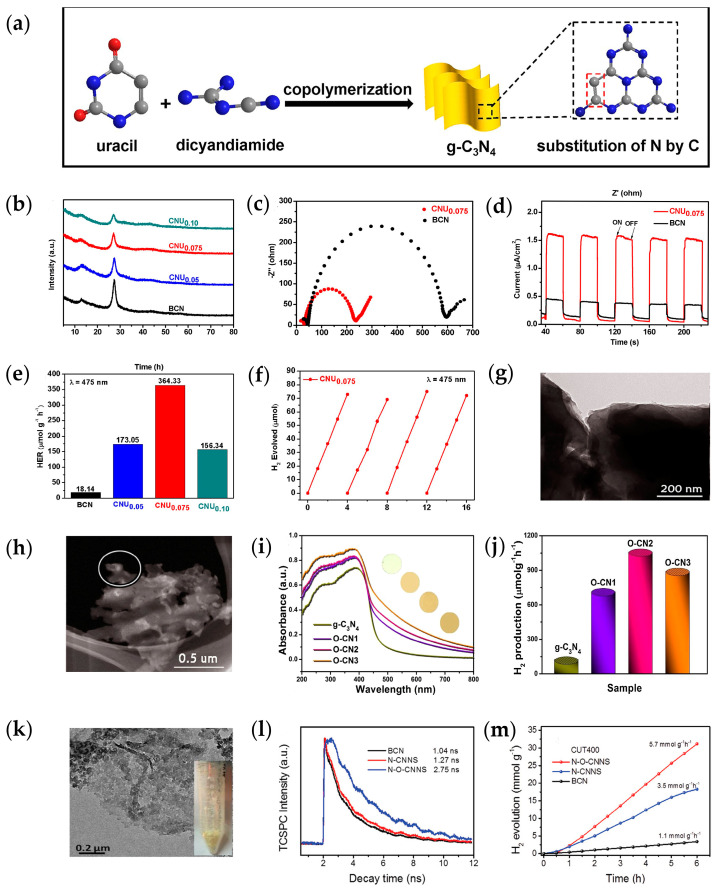
(**a**) The substitution of bridging N in triazine ring of *g*-C_3_N_4_ with carbon atom; (**b**) X-ray photoelectron spectroscopy analysis of CNU; (**c**) Nyquist plots; (**d**) photocurrent response; and (**e**) hydrogen evolution of BCN and CNU; (**f**) stability test of the CNU [85]; TEM images of (**g**) bulk *g*-C_3_N_4_ and (**h**) O/*g*-C_3_N_4_; (**i**) UV–vis light absorption spectra (insets indicate the corresponding color) and (**j**) photocatalytic H_2_ evolution corresponding apparent rate constants of *g*-C_3_N_4_ and O/*g*-C_3_N_4_ [86] (adapted with permission) (**k**) TEM images; (**l**) TCSPC spectra and (**m**) Photocatalytic H_2_ evolution profiles of N-O-CNNS [89] (adapted with permission).

In a recent study, the C atom was used to replace the N atom by copolymerizing the π-electron-rich uracil with dicyandiamide, denoted CNU [85] (Figure 6a). Interestingly, the direct introduction of the C_4_N_2_ ring into *g*-C_3_N_4_ not only maintained the structural stability (Figure 6b) but also reduced the bandgap from 2.75 eV to 2.58 eV, and the dissociation of photogenerated excitons was effectively promoted. An electrochemical impedance spectroscopy study showed that the CNU had a much smaller arc radius than bulk *g*-C_3_N_4_, indicating that adding π bonds to the carbon nitride framework can enhance charge transport (Figure 6c). As a result, CNU samples had about 4–5 times higher photocurrent density under visible light irradiation, compared to bulk *g*-C_3_N_4_ (Figure 6d). More intuitively, doping *g*-C_3_N_4_ with C enhanced the hydrogen production rate to 364.33 μmol·g^−1^·h^−1^, which is about 13 times higher than bulk *g*-C_3_N_4_ (Figure 6e). Despite this moderate performance, the optimal C-doped *g*-C_3_N_4_ collected more sunlight (λ = 750 nm) and also exhibited excellent performance under green (λ = 500 nm) and yellow (λ = 550 nm) light (94.07 and 28.47 μmol·g^−1^·h^−1^, respectively). Moreover, the carbon-rich samples were not significantly inactivated by four successive photocatalytic reactions under continuous blue and visible light irradiation (Figure 6f) and provided excellent photocatalytic stability. Similarly, Liu [86] et al. doped the O atom into *g*-C_3_N_4_ by adding ammonium acetate to melamine in thermal polymerization. Compared with the bulk *g*-C_3_N_4_ (Figure 6g), the O-substituted *g*-C_3_N_4_ exhibited a corrosive-like morphology (Figure 6h). The microstructure of this corrosive state provided more reactive sites, which is conducive to improving the photocatalytic activity. Additionally, the O atom replaces the N in the C-N=C coordination to form the C-O=C bonds in the *g*-C_3_N_4_ lattice [86], allowing *g*-C_3_N_4_ to acquire acceptor levels below the conduction band. As a consequence of adding the acceptor level, its visible light response was broadened to 800 nm (Figure 6i), and the visible light catalytic hydrogen production rate reached 1062.4 μmol·g^−1^·h^−1^ (λ > 420 nm) nine times higher than the original *g*-C_3_N_4_ (Figure 6j). At the same time, the O-doped *g*-C_3_N_4_ maintained a high photocatalytic activity of H_2_ production in each cycle test, and the phase structure of the catalyst did not change after four cycles, which showed good stability and durability.

Moreover, by utilizing the advantages of different doping elements for co-doping, the synergistic effect of the two elements is more conducive to expanding the photo response range of *g*-C_3_N_4_, accelerating charge transfer speed, suppressing the recombination of photogenerated carriers, and significantly improving the photocatalytic performance of *g*-C_3_N_4_. Co-doping can be classified into three types: metal/metal(Mg/Li [87], Na/Fe [88], Co/La [70]), metal/non-metal(Fe/C [71], Mo/S [73], Na/O [72], Bi/S [74]), and non-metal/non-metal(S/P [75], S/C [76]). The hydrogen production efficiency of *g*-C_3_N_4_ significantly increased after co-doping. By copolymerizing melamine with ammonium chloride, N and O co-doping was realized, followed by oxidative etching in air and thermal oxidation-induced exfoliation to obtain excellent ultra-thin *g*-C_3_N_4_ nanosheets (N-O-CNNS) [89]. Owing to the presence of ultra-thin nanosheet structures, the prepared N-O-CNNS exhibit the characteristics of being loose and porous (Figure 6k), providing abundant sites for contact with the reaction medium. The different electronegativity of doped N and O in *g*-C_3_N_4_ nanosheets resulted in the existence of an electric field, and the internal electric field thus enhanced the transient photocurrent density and reduced double-doped photogenerated electron migration resistance, which enabled the rapid transfer of charge during photocatalysis, effectively limiting the recombination of carriers. Meanwhile, the fluorescence emission lifetime of N-O-CNNS was 2.75 ns (Figure 6l), which was 2.1 times that of the single-doped *g*-C_3_N_4_ of N elements(N-CNNS). Consequently, N-O-CNNS exhibited a significant hydrogen production rate of 5.7 μmol·g^−1^·h^−1^ under visible light irradiation, in comparison to 1.6 times that of the N-CNNS (3.5 μmol·g^−1^·h^−1^) (Figure 6m). Moreover, there is no significant attenuation of the H_2_ production rate of N-O-CNNS after five cycles within 18 h, which proves that its high catalytic activity is reproducible, indicating that N-O-CNNS owns high stability under the applied conditions.

### 5.2. Morphology Control

As per functional properties, morphology and internal structure (pore texture/surface area) play an important role in determining its optical and photo-electronic properties. The traditional bulk *g*-C_3_N_4_ prepared usually has a small specific surface area and is heavily stacked between layers. Transforming bulk *g*-C_3_N_4_ into nanostructures (nanotubes, nanorods, nanosheets) or porous structures of different sizes [90] can significantly adjust the surface area, abnormal active sites, and photocatalytic activity trends through morphological control strategies.

#### 5.2.1. Nanostructure Regulation

**Figure 7:**
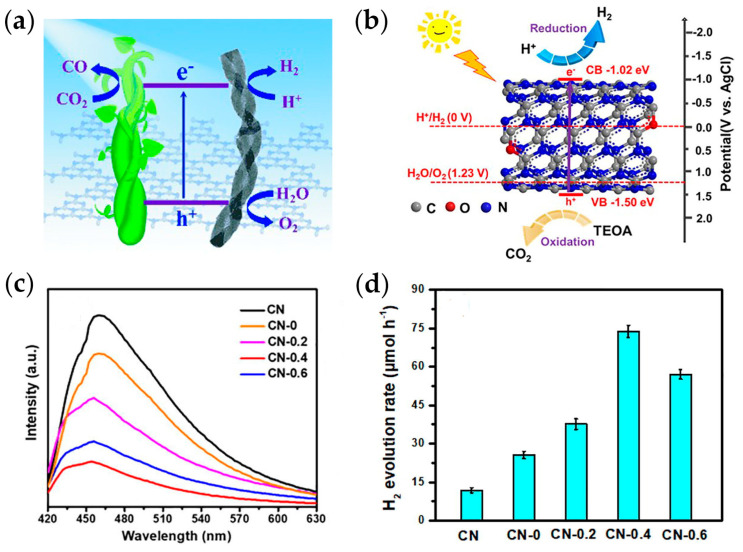
(**a**) *g*-C_3_N_4_ of chiral helical nanorods for photocatalytic water splitting and CO_2_ conversion [91] (adapted with permission); (**b**) photocatalytic hydrogen evolution schematic of OCN nanotubes; (**c**) photoluminescence spectra of OCN nanotubes; (**d**) HER of the bulk CN and OCN samples [92] (adapted with permission).

It is well recognized that constructing nanostructures can greatly improve the functional performance of photocatalysts since the nanoscale surface endows them with a large specific surface area, enhancing the reaction active sites and photo-electronic properties. For example, one-dimensional nanostructures, including nanowires, nanorods, nanotubes, and nanoribbons, can provide fast conduction channels for photogenerated charge transport, thereby improving the photocatalytic performance of *g*-C_3_N_4_. In one pioneering work, Zheng [91] and co-workers fabricated *g*-C_3_N_4_ nanorods with left-handed and right-handed helical nanostructures via nano-casting pathways. They claimed that the chiral helical nanostructure can promote charge separation and mass transfer of *g*-C_3_N_4_, making it a more efficient photocatalyst. To date, the helical *g*-C_3_N_4_ nanorod catalyst unambiguously reveals the photocatalytic capability towards improved hydrogen production rate, CO_2_ to CO conversion, and water oxidation (Figure 7a). On the other hand, a nanotube structure with ultra-thin tube walls and a large specific surface area will be an optimal carrier for transfer charge and provide more adsorption as well as active sites for photocatalytic hydrogen production. As the structure and properties are tuned with certain strategies for the preparation, the fabrication of *g*-C_3_N_4_ nanotubes (CNNTs) gives rise to excellent photocatalytic properties towards the H_2_ evolution rate (Figure 7b). For instance, 1D *g*-C_3_N_4_ (OCN) nanotubes, coupled with oxygen the form C-O-C bonds, were synthesized via a combined hydrothermal and calcination process [92]. The obtained nanotubes had a pore size of 50–70 nm and a wall thickness of 3–4 nm achieved. The results revealed the specific surface area of the nanotubes was 52.9 m^2^·g^−1^, averaging 2.29 times that of bulk *g*-C_3_N_4_. The hollow nanotube structure of OCN provided multiple diffuse reflections for photocatalytic redox reactions, which can significantly improve the utilization capacity of visible light and provide a fast conduction channel for photogenerated charge transport. The unique nanotube structure enabled faster charge transfer and higher photogenerated charge separation efficiency in *g*-C_3_N_4_, which was manifested by a significant increase in photogenerated current density and photoluminescence intensity (Figure 7c). The extended photoresponse range and high charge separation efficiency are beneficial for improving the photocatalytic performance of *g*-C_3_N_4_. As a result, the photocatalytic hydrogen production efficiency of the OCN nanotubes was enhanced to 73.84 μmol·h^−1^, which was 6.24 times that of bulk *g*-C_3_N_4_ (Figure 7d). In addition, OCN exhibited excellent photocatalytic hydrogen evolution stability, maintained a retention rate of 99.1% after three cycles, and did not show degradation of photocatalytic performance after 6 months of storage, proving that OCN is indestructible and reusable.

**Figure 8:**
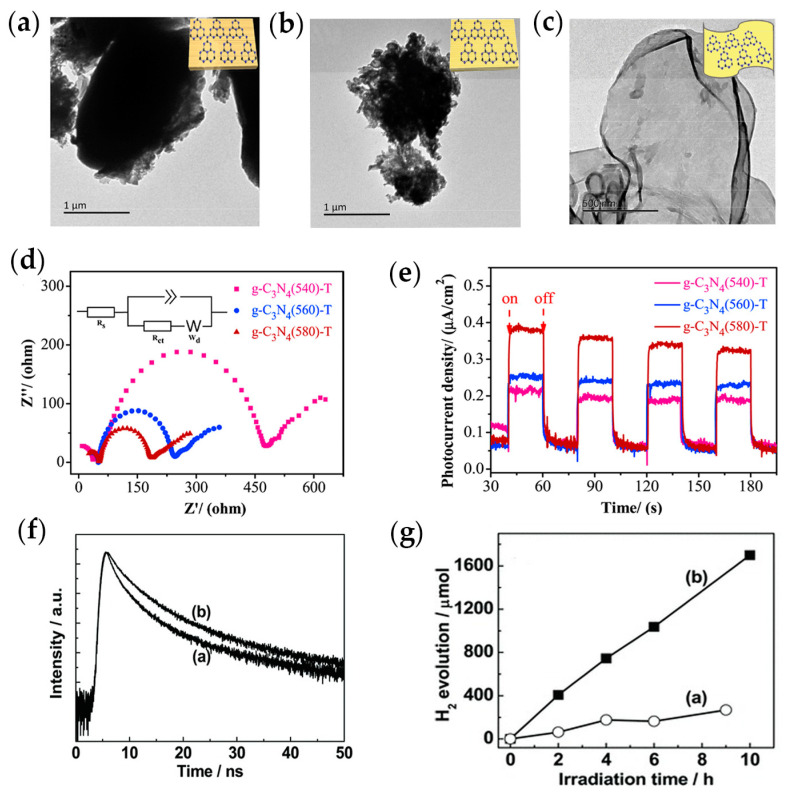
Typical TEM images: (**a**) *g*-C_3_N_4_ (540)-T; (**b**) *g*-C_3_N_4_(560)-T; (**c**) *g*-C_3_N_4_(580)-T; (**d**) EIS of *g*-C_3_N_4_(5X0)-T; (**e**) photocurrent response of *g*-C_3_N_4_(5X0)-T [93] (adapted with permission); (**f**) time-resolved fluorescence decay spectra of *g*-C_3_N_4_ nanosheets: a) bulk *g*-C_3_N_4_, and b) *g*-C_3_N_4_ nanosheets; (**g**) photocatalytic hydrogen evolution of *g*-C_3_N_4_ nanosheets: a) bulk *g*-C_3_N_4_, and b) *g*-C_3_N_4_ nanosheets [94] (adapted with permission).

Another avenue worth exploring is ultra-thin nanosheets structures. In nanostructures, two-dimensional *g*-C_3_N_4_ nanosheets were extensively studied for their photochemical application, bestowing highly accessible functional surfaces with suitable bandgap and structural integrity. The two-dimensional structure significantly shortens the charge transfer distance of the photocatalyst from the inside to the surface, and the carrier transfer rate can be adjusted by changing the thickness of the nanosheet, and when the thickness of the nanosheets is between 2 nm and 0.4 nm, the charge transfer speed accelerates with the decrease in thickness. In addition, the 2D ultra-thin nanosheet structure also provides abundant active sites, which are conducive to light capture and adsorption of reactant molecules. For instance, by controlling the polymerization temperature strategy [93], ultra-thin *g*-C_3_N_4_ nanosheets with an average thickness of 3.5 nm were fabricated, and *g*-C_3_N_4_ of different thicknesses (denoted as *g*-C_3_N_4_-540, *g*-C_3_N_4_-560 and *g*-C_3_N_4_-580) were obtained under heating (heating temperatures of 540 °C, 560° C, 580 °C, respectively) thiourea in the air without adding any template. However, *g*-C_3_N_4_-540 and *g*-C_3_N_4_-560 samples reveal a large agglomerate in microns size (Figure 8a,b). Surprisingly, nanosheets with an average thickness of 3.5 nm were prepared at 580 °C by further increasing the polymerization temperature (Figure 8c). The ultra-thin nanosheet structure promoted the migration and separation rate of photogenerated carriers, which was manifested by a significant decrease in charge transfer resistance (Figure 8d) and a significant increase in photocurrent density (Figure 8e). The *g*-C_3_N_4_ nanosheets with an average thickness of 3.5 nm have a superior visible light photocatalytic H_2_ evolution rate of 1391 μmol·g^−1^·h^−1^. More importantly, *g*-C_3_N_4_-580-T still showed high photostability after five photocatalytic hydrogen evolution cycles (continuous photoreaction for 25 h) without significant inactivation. Compared with fresh samples, there was no significant change in the morphology of *g*-C_3_N_4_-580-T nanosheets. The results show that *g*-C_3_N_4_-580-T holds good visible light response photocatalytic activity and can be effectively applied to the field of photocatalytic hydrogen evolution. In another work, *g*-C_3_N_4_ nanosheets with a thickness of about 2 nm were obtained by etching bulk *g*-C_3_N_4_ via thermal oxidation in air [94]. The optimum anisotropic two-dimensional nanosheets had a high specific surface area of 306 m^2^·g^−1^ compared with bulk *g*-C_3_N_4_ (50 m^2^·g^−1^). Such a large surface area allowed for more reaction sites and efficient charge transfer at reactant molecules for photocatalytic reactions. By thinning the bulk *g*-C_3_N_4_ to the thickness of the nanosheets, the fluorescence lifetime was extended from 4.153 ns to 5.064 ns (Figure 8f), which increased the radiation lifetime of the charge carriers by about 70%, which could effectively increase their probability of participating in the photocatalytic reaction before recombination. The results showed that the nanosheets exhibited stable and efficient hydrogen evolution performance under UV–Vis conditions, with an average hydrogen production rate of 170.5 μmol·h^−1^, which was 5.4 times that of the bulk *g*-C_3_N_4_ (Figure 8g).

#### 5.2.2. Pore Structure Control

In the photocatalyst system, the porous structure can significantly reduce the reflectivity of light, and the multiple reflection scattering of light in the porous structure is conducive to the collection of incident light, and the absorption coefficient of light is increased for photocatalytic reactions [78]. Control of the size, shape, and volume distribution of void spaces is also commonly employed in porous materials for enhanced high photocatalytic hydrogen production. The efficient transfer of light charges to a catalyst surface depends on the quality and quantity of the active sites, which are determined by their surface functional groups and specific surface area, respectively. Therefore, the preparation of porous structures is a quick choice.

**Figure 9:**
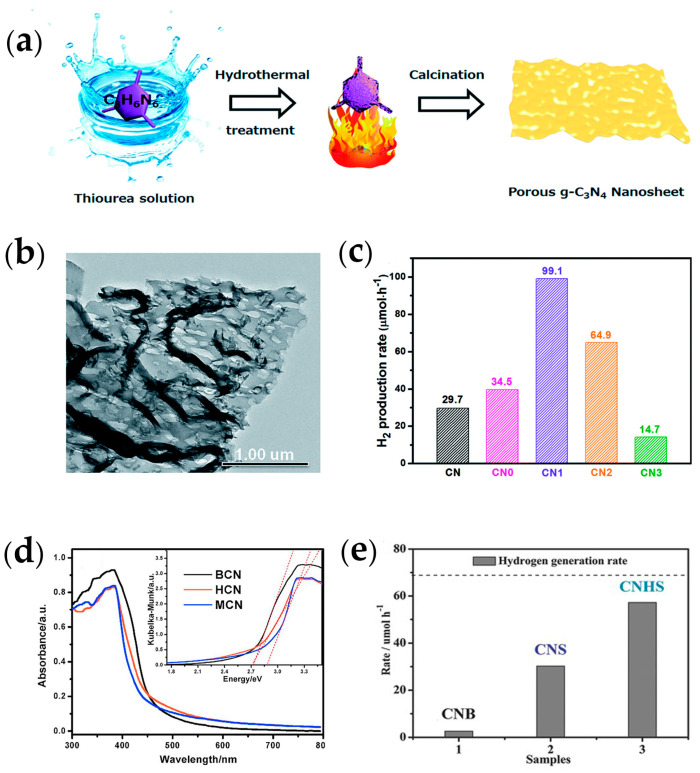
(**a**) Schematic illustration of the preparation of the porous *g*-C_3_N_4_ nanosheets CN1; (**b**) TEM images of CN1; (**c**) photocatalytic H_2_ production rate of CN1 [95] (adapted with permission) (**d**) UV–Vis diffuse reflectance spectra of HCN [96] (adapted with permission); (**e**) photocatalytic H_2_ production rate of CNHS [97] (adapted with permission).

The microporous structure is essential to create a large specific surface area for charge carrier generation, transfer, and transport, exposing more active sites to electrons and ions for photocatalytic redox reactions. Thiourea is a common building block in the fabrication of porous *g*-C_3_N_4_ nanosheets, which regulates the pore structure of *g*-C_3_N_4_ nanosheets by corroding the melamine surface during hydrothermal pretreatment. Huang et al. [95] obtained porous thin *g*-C_3_N_4_ nanosheets (CN1) by traditional thermal polymerization with thiourea-assisted hydrothermal pretreatment of melamine precursors (Figure 9a). Compared to melamine without thiourea, CN1 exhibited thinner and uniform pores (Figure 9b). With a structure consisting of microporous and mesoporous, the CN1 had a pore size of 4.5 nm, and the specific surface area (44.2 m^2^·g^−1^) was 4.9 times that of the original *g*-C_3_N_4_ (9.0 m^2^·g^−1^). The porous structure provides a new possibility for charge mobility, resulting in a significant decrease in CN1 phosphorescent emission intensity and interfacial charge migration resistance. This allows photogenerated electrons and holes to easily migrate to the surface of the catalyst and participate in redox reactions, resulting in high photocatalytic performance. Based on these attributes, the enhanced hydrogen production rate of CN1 (99.1 μmol·h^−1^) was increased by 3.3 times compared to the original *g*-C_3_N_4_ (Figure 9c).

Mesoporous structures are well identified to increase the solar confinement and charge transfer kinetics. A facile synthetic approach towards mesoporous *g*-C_3_N_4_ nanosheets (HCN) [96] was carried out via calcinated dicyandiamide-assisted hydrothermally pretreated precursors. This precursor reforming strategy gave HCN mesopore average pore size of 12.7 nm, a specific surface area of 123.73 m^2^·g^−1^ about 15.4 times that of bulk *g*-C_3_N_4_ (8.04 m^2^·g^−1^), and also significantly enhanced pores size volume (0.392 cm^3^·g^−1^) as comparison bulk *g*-C_3_N_4_ (0.054 cm^3^·g^−1^). Multiple light reflections in the mesoporous texture can enhance the light capture ability to a certain extent, which is conducive to improving the photocatalytic activity. Possessing remarkable light-capturing structural diversity in mesoporous, HCN presented a boosted absorption of 450–800 nm with a remarkable blue shift in absorption edge (Figure 9d). As shown in Figure 9d, HCN retained a bandgap of 2.70 eV with a smaller bandgap, increasing the migration of photogenerated carriers from the valence band guide band. The results showed that the hydrogen evolution rate of HCN with uniformly distributed mesoporous nanosheet structure was 136.9 μmol·h^−1^, which was 15 times that of bulk *g*-C_3_N_4_. After four cycle experiments, the H_2_ evolution rate of HCN did not decay significantly, indicating that its photocatalytic application stability was great.

Considering the vast molecule/ion transportation in its macro-porous structure, the development of macro-porous *g*-C_3_N_4_ with its strikingly improved photogenerated charge transfer and kinetic control reactions was widely investigated for hydrogen production. Very recently, ultra-thin *g*-C_3_N_4_ nanosheets (CNHS) with macro-porous structures were synthesized using facile post-thermal annealing in an air atmosphere by thermal annealing [97]. Based on in situ experimental investigation, CNHS exhibited porosity with a few nm to 100 nm. The specific surface area of CNHS was 277.98 m^2^·g^−1^, 26 times higher than that of bulk *g*-C_3_N_4_ (10.89 m^2^·g^−1^), and photoluminescence lifetime was calculated to be 1.54 times that of bulk *g*-C_3_N_4_. The enhancement of the optoelectronic performance of CNHS can be attributed to the fact that the macroporous structure greatly promotes mass transfer, increasing the photogenerated charge mobility and the probability of their participation in the photocatalytic reaction before recombination. The results showed that the average hydrogen production rate of CNHS was 57.20 μmol·h^−1^ (Figure 9e), about 22.24 times enhancement over that of bulk *g*-C_3_N_4_ (2.57 μmol·h^−1^). Moreover, the photocatalytic hydrogen production rate of CNHS remained unchanged after four cycles, showing excellent cycling stability. This will be beneficial to the development of practical applications of *g*-C_3_N_4_ photocatalysts.

### 5.3. Construction of g-C_3_N_4_ Heterostructure

Photogenerated charge carriers will recombine inside or on the surface of *g*-C_3_N_4_, and a large amount of energy will be dissipated in the form of heat or light, which greatly limits the photocatalytic efficiency of *g*-C_3_N_4_. Since photogenerated charge carriers can exist at the interface of another conductor, semiconductor, or cocatalyst, the efficiency of photogenerated electron–hole separation can be improved by constructing suitable heterojunctions [98]. According to the relative arrangement of HOMO and LUMO energy of the organic components, heterojunctions are mainly divided into three types: type I (straddling gap type), type II (staggered gap type) and type III (broken-gap type) [99]. Since the photogenerated electrons and holes in type I heterojunction are confined to the low redox potential region, the photogenerated carriers cannot be effectively separated, so it is not described. At present, the construction of heterojunctions between *g*-C_3_N_4_ and appropriate semiconductors has been proven to be effective in improving the efficiency of photogenerated charge separation and is widely used to improve the efficiency of photocatalytic hydrogen production.

**Figure 10:**
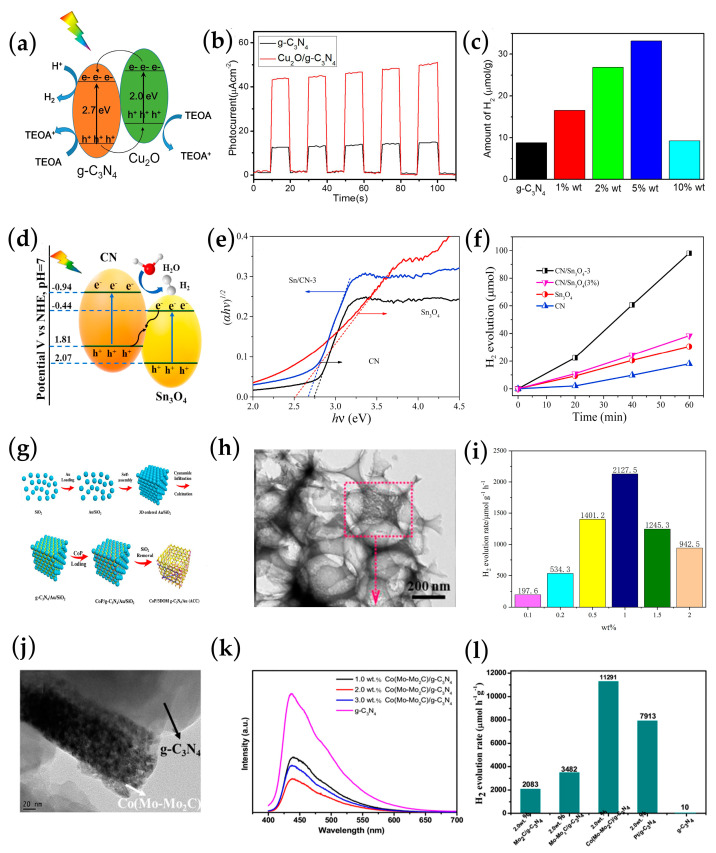
(**a**) Diagram of the chemical mechanism of *g*-C_3_N_4_/Cu_2_O; (**b**) photocurrent responses curves of *g*-C_3_N_4_/Cu_2_O; (**c**) photocatalytic hydrogen evolution of prepared photocatalysts under visible light [100] (adapted with permission); (**d**) diagram of the chemical mechanism of *g*-C_3_N_4_/Sn_3_O_4_; (**e**) ultraviolet absorption spectra of *g*-C_3_N_4_ and *g*-C_3_N_4_/Sn_3_O_4_; (**f**) photocatalytic hydrogen evolution of prepared photocatalysts under visible light [101] (adapted with permission); (**g**) synthetic route of *g*-C_3_N_4_/Au; (**h**) Transmission Electron Microscope of *g*-C_3_N_4_/Au; (**i**) photocatalytic hydrogen evolution of *g*-C_3_N_4_/Au [102] (adapted with permission); (**j**) HR-TEM images; (**k**) photoluminescence spectra; and (**l**) photocatalytic H_2_ production rate of Co (Mo-Mo_2_C)/*g*-C_3_N_4_ [108] (adapted with permission).

Type II heterojunctions based on *g*-C_3_N_4_ were shown to effectively promote carrier separation by inducing band bending and the formation of an internal electric field [99]. As shown in this report, the *g*-C_3_N_4_/Cu_2_O type II heterojunctions hybrid material prepared in situ significantly showed drastic enhanced photocatalytic activity toward hydrogen production [100], the electrons and holes are spatially separated, and the electrons flow into the semiconductor with more positive LUMO (*g*-C_3_N_4_), while the hole flows to the semiconductor with more negative HOMO (Cu_2_O) (Figure 10a). This staggered gap-type band structure is effective for the separation of electron–hole pairs. Noticeably, a drastic increase change in g-C_3_N_4_/Cu_2_O photocurrent intensity indicates that the separation and transfer of photogenerated electron–hole pairs at the tight interface between *g*-C_3_N_4_ and Cu_2_O are effectively improved, making a promise for the separation and transfer of charge (Figure 10b). Remarkably, the optimal Cu_2_O doping content (5 wt%-Cu_2_O/*g*-C_3_N_4_) photocatalyst exhibited hydrogen production of 33.2 μmol·g^−1^·h^−1^, which is about four times higher than pure *g*-C_3_N_4_ (Figure 10c). And the amount of hydrogen evolution of g-C_3_N_4_/Cu_2_O in the three cycle experiments was almost the same, indicating that the photocatalyst has good stability for hydrogen production and has practical application value.

Recently, the Z-scheme recombination mechanism of heterojunction was widely used to facilitate charge transfer separation efficiency. It is unambiguously revealed that the Z-scheme recombination mechanism of heterojunction not only facilitates charge transfer separation efficiency but also maintains a strong photo-redox capacity. A prime example is the successful preparation of 2D *g*-C_3_N_4_/2D Sn_3_O_4_ organic/inorganic Z-type heterojunctions via calcination strategy [101] (Figure 10d). With these, a heterojunction formation in Sn_3_O_4_, and *g*-C_3_N_4_, revealed optimal photogenerated holes, which would enhance the lifetime of the photoluminescence charge carrier. The electrons were positioned at the high conduction band (CB) of *g*-C_3_N_4_, while holes were positioned at the low Sn_3_O_4_ to control the strong redox capacity. By utilizing terephthalic acid as a probe molecule for the detection of the active •OH radicals, the chemiluminescence method demonstrated that the maximum photoluminescence signal appeared at 425 nm, and its increasing intensity with light time exhibited that the photodegradation reaction produced •OH free radicals. Thanks to the band arrangement of the Z-mode, the charge was efficiently transferred, resulting in a strong redox potential, which enabled *g*-C_3_N_4_ with a low valence band (VB) value to directly generate hydroxyl groups (•OH) after the combination of Sn_3_O_4_. With the Z-scheme electron transfer interface, the absorption spectrum of *g*-C_3_N_4_/Sn_3_O_4_ could be combined, achieving the full spectral response (300–800 nm) (Figure 10e). Meanwhile, g-C_3_N_4_/Sn_3_O_4_ exhibited much lower charge transfer resistance than regular Sn_3_O_4_, which improved carrier separation. More significantly, the photocurrent response value achieved 3.0 × 10^−3^ mA, which was 5.6 times higher than *g*-C_3_N_4_ and 2.9 times that of Sn_3_O_4_. It is indeed a remarkable achievement of photocatalytic H_2_ production, the hydrogen release rate of the optimal Sn_3_O_4_ doped content (3 wt%) was 98.07 μmol·h^−1^, which was 5.4 times higher than that of *g*-C_3_N_4_ (18.07 μmol·h^−1^) (Figure 10f). It is worth noting that g-C_3_N_4_/Sn_3_O_4_ basically maintained 88% of the initial photocatalytic hydrogen evolution activity after the fifth cycle (10 h), and the composition of the used sample was consistent with that of the unused sample. It is proved that g-C_3_N_4_/Sn_3_O_4_ possesses outstanding repeatability and stability in the process of photocatalytic hydrogen evolution, so it has great potential as an efficient and stable photocatalyst.

Another way to improve the photocatalytic activity of *g*-C_3_N_4_ is to couple with co-catalysts, as they can provide active sites and capture photogenerated carriers. Noble metal cocatalysts including Au, Ag, Pt, and Pd particularly Au, are hybridized to g-C_3_N_4_ as an optical trap due to its strong special surface plasmon resonance effect (SPR), which improves visible light absorption and charge carrier separation. For instance, Au nanoparticles (AuNPs) incorporated with *g*-C_3_N_4_ (PCN) make them promising for the macro-porous frame with 3-dimensional order to form 3DOM *g*-C_3_N_4_/Au (ACN) [102] (Figure 10g). Incorporating AuNPs significantly improved specific surface area (SSA) (20.02 m^2^·g^−1^) and pore ratio (0.19 cm^3^·g^−1^) (Figure 10h) as comparison small SSA and pore volume of PCN (12.79 m^2^·g^−1^, 0.09 m^3^·g^−1^). Because of the SPR effect of the AuNPs, ACN displayed enhanced visible light absorption till 550 nm. In the 3D *g*-C_3_N_4_ framework loaded with a single co-catalyst Au, the photoluminescence fluorescence of ACN was partially quenched, which enabled charge transfer to a certain extent. As a result, ACN samples produced hydrogen at 2127.5 μmol·g^−1^·h^−1^ (Figure 10i), approximately 111.4 times that of PCN. The ACN samples also hold outstanding stability and reusability, and the hydrogen production activity of the samples remained stable compared to its initial Hydrogen Evolution Reaction (HER) value. This novel nanostructured photocatalyst is expected to be a candidate material for photocatalytic hydrogen production.

Noble metal cocatalysts hold excellent electron extraction ability and have a decisive impact on the photocatalytic hydrogen production activity of *g*-C_3_N_4_, which has been widely studied. However, the high cost severely limits its wide range of technical applications; therefore, low-cost transition metal-based are gradually being widely studied to replace noble metal-based cocatalysts. Transition metal cocatalysts, such as transition metal phosphide (CoP [103], Co_2_P [104]), transition metal sulfides (CoS_2_ [66]_,_ WS_2_ [105], MoS_2_ [106]), transition metal carbides (Ni_3_C, Co (Mo-Mo_2_C) [107]), etc., are potential alternatives to noble metal cocatalysts due to their low cost, high storage capacity, and strong electron capture capacity. Among them, transition metal phosphide has the advantages of high activity, good stability and easy preparation. Transition metal sulfides have an adjustable surface structure, and their polar sulfur surfaces have a strong affinity for hydrogen protons. Transition metal carbides have excellent metallic properties and thermal stability, as well as strong corrosion resistance in acidic environments. In recent years, transition metal carbides have been considered proton reduction sites, enabling rapid diffusion of electrons from *g*-C_3_N_4_ to metal carbides [18]. These advantages make transition metal cocatalysts promising candidates for precious metal cocatalysts in the field of photocatalytic hydrogen production. In one study, Zheng [108] et al. doped Co into Mo-Mo_2_C, and used ultrasound assisted deposition method to load Co (Mo-Mo_2_C) on the surface of *g*-C_3_N_4_ to construct a Co (Mo-Mo_2_C)/*g*-C_3_N_4_ photocatalyst. The study found that Co (Mo-Mo_2_C) was uniformly loaded on the layered *g*-C_3_N_4_ surface in the form of nanorods (Figure 10j). The nanorod-shaped Co (Mo-Mo_2_C) cocatalyst provided a passivation effect on most of the surface states of *g*-C_3_N_4_, which can significantly decrease the charge recombination rate and increase the carrier transfer rate (Figure 10k), catalyzing the proton reaction, so that the electrons can be used for photocatalytic hydrogen production. When the Co (Mo-Mo_2_C) loading was 2.0 wt%, the hydrogen production reached a maximum of 11,291 μmol·g^−1^·h^−1^, which is 3.2 times higher than that of the undoped one (Figure 10l). And the photocatalytic performance of the 2.0 wt% Co (Mo-Mo_2_C) sample was not significantly reduced under 40 h of light, and the addition of an appropriate amount of TEOA could restore the activity, which had good stability and reusability. The results show that the Co (Mo-Mo_2_C) cocatalyst can effectively improve the photocatalytic activity of *g*-C_3_N_4_, making it a potential candidate to replace the precious metal cocatalyst in the field of photocatalytic hydrogen production.

## 6. Conclusions and Prospectives

As an organic semiconductor in photocatalysis, *g*-C_3_N_4_ distinguishes itself in the research of hydrogen generation owing to its desirable light absorption, high thermal and chemical stability, and high economic efficiency. Nonetheless, easy recombination and low quantum efficiency of photoexcited charge carriers constrain further larger-scale industrial applications. In this work, a survey of modification strategies of high-performance photocatalysts based on *g*-C_3_N_4_ is laid out, including elemental doping, morphology regulation control, and construction of heterojunctions between *g*-C_3_N_4_ and appropriate semiconductors.

1. Elemental doping alters the energy band structure and surface properties of *g*-C_3_N_4_ through orbital hybridization, enlarges the light-absorbing capacity of *g*-C_3_N_4_, and facilitates the effective separation of photogenerated carriers, thus conferring excellent photocatalytic performance of *g*-C_3_N_4_. Whether it be metal, non-metal, or co-doping methods, all have achieved significant performance enhancements. However, it should be highlighted that the benchmarks for gauging these improvements are not standardized across studies. For instance, some research has reported a 10,000-fold surge in hydrogen production rates post-doping, while other studies have focused on achieving higher overall hydrogen production yields or enhanced apparent quantum efficiencies. Despite these advancements, the quest for a material design that embodies the ultimate combination of qualities remains ongoing.
2. The morphology regulation control (e.g., nanotubes, nanorods, nanosheets, or porous structure) can significantly increase the surface area, and reaction active sites and enhance the photocatalytic activity. While the aforementioned nanostructures have demonstrated the potential to markedly improve performance, their effectiveness is still primarily benchmarked against bulk *g*-C_3_N_4_. The current state of research exhibits a deficiency in systematic comparative analyses that span the spectrum from 0D to 3D nanostructures within the same material system. Additionally, there is a notable absence of discourse on the specific application contexts where these nanostructures would be most advantageous. This gap in the existing research limits the comprehensive understanding of how nanostructures can be tailored to meet the demands of various photocatalytic applications.
3. By constructing suitable heterojunctions to allow photogenerated charge carriers to exist at the interface of another conductor, semiconductor, or catalyst, inhibiting their recombination inside or on the surface of *g*-C_3_N_4_ and reducing energy loss, the efficiency of photogenerated electron–hole separation can be effectively improved, thus enhancing the efficiency of photocatalytic hydrogen production. However, the introduction of a second material can sometimes compromise the overall performance stability of the photocatalyst, leading to degradation under prolonged use. Concurrently, the additional steps required to create heterostructures can increase the cost of production, and there must be a balance between the cost and the efficiency gains achieved.

In summary, this review provides a comprehensive overview of the most recent advancements in modifying *g*-C_3_N_4_ for photocatalytic hydrogen production under visible light, highlighting the efficacy of various strategies in enhancing performance. While the field has witnessed remarkable progress, much of the research is still in the theoretical validation stage. It is marked by a variety of performance metrics that are yet to be standardized into a cohesive framework. The current body of work has predominantly generated data applicable to laboratory conditions, focusing on hydrogen evolution, without concrete guidelines for transitioning to practical industrial applications.

Through this article, it is evident that the current research focus is on enhancing the separation and migration efficiency of photogenerated charge carriers, as well as increasing the specific surface area and the number of active sites. In addition to these, *g*-C_3_N_4_ still faces issues such as crystallinity problems, limited light absorption range, and the need for improved stability and long-term performance. Traditionally synthesized *g*-C_3_N_4_ materials, prepared through high-temperature calcination, often exhibit poor crystallinity and a significant number of internal and surface defects. These imperfections can act as recombination centers for photogenerated charge carriers, leading to rapid electron–hole recombination and a consequent decrease in photocatalytic performance. This issue can be addressed by investigating novel synthetic approaches, such as the combination of soft and hard templating methods, to circumvent the shortcomings of individual synthetic techniques. By employing these advanced strategies, it is possible to achieve *g*-C_3_N_4_ materials with enhanced crystallinity and reduced defects, thereby improving their photocatalytic efficiency. The absorption spectrum of graphitic carbon nitride (*g*-C_3_N_4_) is constrained to the visible light region, which diminishes the overall solar energy utilization efficiency. Prolonged exposure to photocatalytic conditions can lead to structural degradation and performance decline of *g*-C_3_N_4_, thereby compromising its stability for long-term applications. These attributes are intrinsically linked to the strategies employed in its synthesis and subsequent modification strategies.

Furthermore, existing strategies have typically adopted only parts of the aforementioned methods, missing out on the potential for controlled synergistic enhancements that a comprehensive, multi-strategy approach could offer. This shortfall underscores the necessity for developing more integrated and synergistic strategies. Such an approach is essential for significantly boosting the photocatalytic capabilities of *g*-C_3_N_4_, steering the research from theoretical exploration toward impactful, real-world applications.

## Figures and Tables

**Table 1:** Summaries of the commonly studied element-doped *g*-C_3_N_4_ photocatalysts and the corresponding PHE activities.

Doping Type	Doping Element	Co-Catalyst	Morphology	Light Source	R (H_2_) (μmol·g^−1^·h^−1^)	R (H_2_) of *g*-C_3_N_4_ (μmol·g^−1^·h^−1^)	Apparent Quantum Efficiency (AQE%)	Application	Ref. (Year)
Transition Metal	Ni	0.5wt%Pt	Ni nanoparticles	500 W Hg lamp	1990	738	N/A	H_2_ evolution	2023 [53]
	Ni	3wt%Pt	Nanosheets 10 nm	300 W Xe lamp, >400 nm	155.71	98.67	N/A	H_2_ evolution, Mo degradation	2019 [54]
	Co	MoS_2_	2D-irregular wrinkled sheet	300 W Xe lamp	3193	76	16.62 (370 nm)	H_2_ evolution	2021 [55]
	Cu	N/A	Nanosheets	300 W Xe lamp	266	14	N/A	H_2_ evolution	2023 [56]
	Cu	N/A	Nanosheets	light-emitting diode, 427 nm	3774.35	1116.07	1.34 (427 nm)	H_2_ evolution	2021 [57]
Alkali Metal	Na	3wt%Pt	Nanowires 100–150 nm	300 W Xe lamp, >420 nm	1740	49.7	N/A	H_2_ evolution	2020 [58]
	K	3wt%Pt	Nanosheets 1–2 nm	300 W Xe lamp, >420 nm	1292.525	71.295	N/A	H_2_ evolution, Rhodamine B degradation	2024 [59]
Non-Metal	C	N/A	C-doped Nanowires, nanosheets	300 W Xe lamp, >420 nm	836	102	N/A	H_2_ evolution, Pollutants degradation	2021 [60]
	C	1.2wt%Pt	Porous sheet structure	300 W Xe lamp	13,169.0	880.82	2.91 (430 nm)	H_2_ evolution	2022 [61]
	N	3wt%Pt	Nanosheets 2.5 nm	300 W Xe lamp, >420 nm	1686.4	259.16	6.2 (420 nm)	H_2_ evolution	2020 [62]
	N	Ni	Bamboo-like carbon nanotubes	300 W Xe lamp, >420 nm	1050.4	21.4	8.12 (420 nm)	H_2_ evolution, 4-nitro-phenol degradation	2023 [63]
	P	2wt%Pt	Hollow ball structure	300 W Xe lamp, >420 nm	9653	N/A	N/A	H_2_ evolution	2021 [64]
	P	Ni (II)	2D-stacked nanosheets	300 W Xe lamp, >420 nm	1213	117	9.7 (420 nm)	H_2_ evolution	2021 [65]
	S	CoS_2_	Nanosheets	300 W Xe lamp, >420 nm	577.5	N/A	1.08 (420 nm)	H_2_ evolution	2020 [66]
	S	3wt%Pt	Porous layered structure	300 W Xe lamp, >420 nm	1404	60	3.3 (420 nm)	H_2_ evolution	2020 [67]
	B	3wt%Pt	Nanosheets	300 W Xe lamp, >420 nm	1639.29	901.35	N/A	H_2_ evolution, NO removal	2022 [68]
	O	1wt%Pt	Crystalline/ amorphous mosaic nanohomo junction	300 W Xe lamp, >420 nm	4073.2	47.3	26.9 (420 nm)	H_2_ evolution	2021 [69]
Metal/Metal	Co/La	N/A	Nanosheets	300 W Xe lamp	1000	400	N/A	H_2_ evolution	2021 [70]
Metal/ Non-Metal	Fe-C	N/A	2D-structure	300 W Xe lamp	2412.38	407.56	N/A	H_2_ evolution	2024 [71]
	Na-O	N/A	Nanorods	300 W Xe lamp, >400 nm	7460	88.8	N/A	H_2_ evolution	2021 [72]
	Mo-S	N/A	Nanosheets	300 W Xe lamp, >420 nm	294	N/A	N/A	H_2_ evolution	2021 [73]
	Bi-S	N/A	Porous 2D-nanosheets structure	300 W Xe lamp, >420 nm	1139	455.6	N/A	H_2_ evolution, Tetracycline degradation	2022 [74]
Non-Metal/ Non-Metal	S/P	N/A	Nanosheets	300 W Xe lamp, >420 nm	103.88	52.14	N/A	H_2_ evolution, Rhodamine B degradation	2024 [75]
	S/C	N/A	Hierarchical microtubules filled with nanosheets	300 W Xe lamp, >420 nm	4868	N/A	N/A	H_2_ evolution	2024 [76]
